# Merozoite Proteins Discovered by qRT-PCR-Based Transcriptome Screening of *Plasmodium falciparum*

**DOI:** 10.3389/fcimb.2021.777955

**Published:** 2021-12-09

**Authors:** Nan Hou, Shanshan Li, Ning Jiang, Xianyu Piao, Yu Ma, Shuai Liu, Qijun Chen

**Affiliations:** ^1^ NHC Key Laboratory of Systems Biology of Pathogens, Institute of Pathogen Biology, Chinese Academy of Medical Sciences & Peking Union Medical College, Beijing, China; ^2^ Key Laboratory of Livestock Infectious Diseases in Northeast China, Ministry of Education, Key Laboratory of Zoonosis, College of Animal Science and Veterinary Medicine, Shenyang Agricultural University, Shenyang, China; ^3^ The Research Unit for Pathogenic Mechanisms of Zoonotic Parasites, Chinese Academy of Medical Sciences, Shenyang, China

**Keywords:** invasion, malaria, merozoite antigen, *Plasmodium falciparum*, transcriptome

## Abstract

The development of malaria vaccines and medicines depends on the discovery of novel malaria protein targets, but the functions of more than 40% of *P. falciparum* genes remain unknown. Asexual parasites are the critical stage that leads to serious clinical symptoms and that can be modulated by malaria treatments and vaccines. To identify critical genes involved in the development of *Plasmodium* parasites within erythrocytes, the expression profile of more than 5,000 genes distributed across the 14 chromosomes of the PF3D7 strain during its six critical developmental stages (merozoite, early-ring, late-ring, early trophozoite, late-trophozoite, and middle-schizont) was evaluated. Hence, a qRT-PCR-based transcriptome of the erythrocytic developmental process of *P. falciparum* was revealed. Weighted gene coexpression network analyses revealed that a large number of genes are upregulated during the merozoite release process. Further gene ontology analysis revealed that a cluster of genes is associated with merozoite and may be apical complex components. Among these genes, 135 were comprised within chromosome 14, and 80% of them were previously unknown in functions. Western blot and immunofluorescence assays using newly developed corresponding antibodies showed that some of these newly discovered proteins are highly expressed in merozoites. Further invasion inhibition assays revealed that specific antibodies against several novel merozoite proteins can interfere with parasite invasion. Taken together, our study provides a developmental transcriptome of the asexual parasites of *P. falciparum* and identifies a group of previously unknown merozoite proteins that may play important roles in the process of merozoite invasion.

## Introduction

Although malaria management has yield outstanding achievements in the past 20 years, there has been no significant progress in reducing global malaria incidence in recent years as reported in the World Malaria Report 2020 published by the World Health Organization (World Health Organization, 2020). Continued efforts have been made to control malaria, and malaria eradication has been put forward by the Lancet Commission (Feachem et al., 2019). However, the currently available diagnostic and treatment tools and approaches are still insufficient to achieve malaria eradication worldwide.

The development of effective malaria vaccines and drugs requires the discovery of novel protein targets. Since the completion of the first draft sequence of the *Plasmodium falciparum* 3D7 genome in 2002 (Gardner et al., 2002), genomic research on malaria parasites has advanced rapidly. Indeed, publicly available databases currently provide genomic information of not only the important species that infect humans (Gardner et al., 2002; Pain et al., 2008; Bright et al., 2012; Rutledge et al., 2017), as well as of primates, rodent, and avian parasites (Tachibana et al., 2012; Otto et al., 2014a; Otto et al., 2014b; Bohme et al., 2018). Overall, *P. falciparum* sequencing promoted by the Malaria Genome Project has provided the foundation for the discovery of numerous malaria antigens, but the function of more than 40% of its genes remain unknown (Gardner et al., 2002). Hence, since then, based on the genetic and genomic information of *P. falciparum*, various studies were performed to fill this knowledge gap. Specifically, single-cell sequencing has allowed high-resolution mapping of developmental processes, cellular diversity, cell-to-cell variation, and even analysis of individual-level variation between parasites across their full life cycle (Poran et al., 2017; Trevino et al., 2017; Howick et al., 2019). Moreover, studies on genomic reductions have identified some essential genes of *Plasmodium* for optimal growth of asexual blood stages *in vitro* (Bushell et al., 2017; Zhang et al., 2018). These studies provided valuable information about the parasite genes; nonetheless, the proteins expressed at the parasite stages still need to be scrutinized for their functions.

The erythrocytic stage of *Plasmodium* is critical for its successful proliferation and transmission (Cowman et al., 2016). The asexual parasites lead to serious clinical symptoms, whereas the pre-erythrocytic stages may not cause symptoms. The majority of malarial antigens expressed during the early pre-erythrocytic stages are not expressed during the erythrocytic stage of infection (Riley and Stewart, 2013), leaving individuals immunized with a pre-erythrocytic vaccine still susceptible to erythrocytic stage infection, especially in areas of intense transmission where entomological inoculation rates may exceed 300 infective bites per year. Therefore, it is imperative to develop a vaccine that incorporates components that target parasite developing within erythrocytes. In particular, merozoites are the only erythrocytic stage parasite form that is directly exposed to the host circulation. Merozoite antigens are presented on the surface of infected erythrocytes and are released upon contact host erythrocytes, thereby representing direct targets of naturally acquired immunity. Merozoite invasion involves multiple interactions and events, but much more still need to learn about the kinetics of merozoite invasion (Beeson et al., 2016). It is indeed possible that protective immunity will require the induction of immune responses to hundreds of target proteins (Bustamante et al., 2017; Favuzza et al., 2017), but only a few proteins have been used in the existing vaccine trials. Thus, the identification of novel merozoite antigens will provide a brighter prospect for the development of malaria vaccines.

Currently, the most common approach to find antigens for asexually stage vaccines depends on serological methods. However, our previous work revealed that most immunogenic epitopes were predominantly located in the low-complexity regions of the parasite proteins, and these epitopes may drive immune responses away from the functional domains of *Plasmodium* pathogenic proteins (Hou et al., 2020). Thus, the present study aimed to identify novel malaria antigens based on their functions. We collected six critical developmental stages (merozoite, early-ring, late-ring, early trophozoite, late-trophozoite, and middle-schizont) of the asexual parasites of the *P. falciparum* 3D7 strain (PF3D7). The transcriptional expression of more than 90% of the genes comprised on the 14 chromosomes of these parasites was successfully detected by real-time quantitative polymerase chain reaction (qRT-PCR). Overall, a qRT-PCR-based transcriptome of *P. falciparum* is herein described. Moreover, weighted gene coexpression network and gene ontology analyses were performed to explore genes associated with merozoites.

## Materials and Methods

### Ethical Statement

All experiments using human samples were performed in accordance with the tenets of the Declaration of Helsinki. Sera samples were donated by healthy volunteers and *P. falciparum* infected patients. Human red blood cells (RBCs) were obtained from three healthy volunteers. Informed consents were obtained from all individuals involved in the study, and all information pertaining to the individuals was anonymized. Studies in humans were reviewed and approved by the Ethical Committee of the Chinese Academy of Medical Sciences (approval no. IPB-2016-2).

### *Plasmodium falciparum* Culture and Collection

PF3D7 strain was cultured and synchronized as previously described (Trager and Jensen, 2005). Briefly, the parasites were routinely grown with human RBCs at 5% hematocrit in a candle jar at 37°C. Malaria culture media consisted of RPMI 1640 culture medium (Invitrogen, Waltham, MA, USA) supplemented with 25 mM HEPES, 25 mM NaHCO_3_, 10 mM glucose, 180 μM hypoxanthine, 2 mM L-glutamine, 20 μg/mL gentamicin, and 0.5% Albumax II (Gibco, Waltham, MA, USA), adjusted to pH 7.4. Parasitemia was detected by Giemsa staining of thin blood smears. The growth of the parasites was synchronized in the early stages by two rounds of treatment with 5% (w/v) d-sorbitol (Sigma-Aldrich, St. Louis, MO, USA) at 48 h intervals. Parasites were collected with eight-hour intervals. The parasites were collected at about 8, 16, 24, 32, 40 and 48 hours post infection (hpi) to obtain early rings, late rings, early trophozoites, late trophozoites, middle schizonts and merozoites. The parasites were collected by centrifugation at 1,500 rpm for 5 min, and the pellets were collected for nucleic acid preparation. The homogeneity of parasites in each timepoint was examined by Giemsa staining, and more than 1,000 parasites from continuous fields of vision were counted to determine the percentage of parasites of indicated stage. The percentages greater than 98% are considered as qualified homogeneity. Thin blood smears were prepared with the culture 46-48 hpi containing free merozoites and schizonts for immunofluorescence assay. For better understanding of the workflow, please refer to the flowchart of the **Supplementary Figure 1**.

### Human Samples

Twenty patients with falciparum malaria (FM) were recruited. All patients experienced fever (> 37.5°C). Blood samples were microscopically examined using Giemsa-stained blood smears and documented as *P. falciparum* infection, and further confirmed by nested PCR (Kimura and Liu, 1997). Patients were recruited in Henan and Yunan, China, from September 2011 to January 2012. Serum samples from all patients were obtained before the patients received treatment. Sera samples from 20 healthy individuals were collected in Beijing, China, from September 2011 to January 2012, and were used as controls. Additional information about the individuals involved in this study is presented in **Supplementary Table 1**.

### Nucleic Acid Preparation

Total RNA was extracted from parasites at different developmental stages using the RNeasy Mini kit (Qiagen, Hilden, Germany), and the genomic DNA contaminants were completely removed from the RNA samples using the TURBO DNA-free Kit (Ambion, Austin, TX, USA) according to the manufacturer’s instructions. RNA quantification and quality control were conducted using a Nanodrop ND-1000 spectrophotometer (Thermo Fisher Scientific, Waltham, MA, USA) and denaturing agarose gel electrophoresis. Total RNA (1 μg) was reverse transcribed into cDNA using the SuperScript III Reverse Transcriptase Kit (Invitrogen) according to the manufacturer’s instructions. For each cDNA synthesis reaction, a control reaction without reverse transcriptase was performed with identical amounts of template RNA. Genomic DNA was prepared using the DNeasy Blood & Tissue Kit (Qiagen) with RNase treatment before the last step of extraction.

### qRT-PCR and Data Analysis

Genomic sequences of PF3D7 were obtained from PlasmoDB (https://plasmodb.org/plasmo/) in September 2015 and used as templates for primer design using Primer BLAST (https://www.ncbi.nlm.nih.gov/tools/primer-blast/). A total of 5,510 gene sequences were derived from the genomic sequences of chromosomes 1–14, which were trimmed down to 5,458 sequences after redundancy removal using cd-hit-v4.3 software (http://bioinformatics.ljcrf.edu/cd-hi/) with coverage standard ≥ 90% and identity ≥ 80%. The specificity of the primers was confirmed by blasting all genomes of Homo sapiens and *P. falciparum* 3D7 (taxid, 36329). All primers of PF3D7 genes used for qRT-PCR are listed in **Supplementary File 1**. Seryl-tRNA synthetase was used as an endogenous control (forward primer: ATGGAACAATGGTAGCTGCAC, reverse primer: GGGCGCAATTTTTCAGGAACTA). The concentrations of the primers for the targets and reference genes were optimized using serially diluted PF3D7 genomic DNA. The cDNA, used for final qRT-PCR detection, was equally mixed with cDNA from three batches of PF3D7 parasites and adjusted to 500 ng/mL, and triplicate reactions, detecting the expression of β-tubulin (forward primer: CGTGCTGGCCCCTTTG, reverse primer: TCCTGCACCTGTTTGACCAA) with merozoite cDNA, were arranged on each plate for standard controls, whereas reactions without cDNA for mixed primers and duplicate reactions were arranged as negative controls. The well arrangement on the 96-well plates was showed in **Supplementary Figure 1B**. Reactions were performed in technical triplicates on the 7500 Real-time PCR System (Applied Biosystems, Waltham, MA, USA) using Brilliant II SYBR Green QPCR Master Mix (Agilent Technologies, Santa Clara, CA, USA) according to the manufacturer’s instructions. Each reaction was performed in a final volume of 20 μL containing 10 μL 2× Brilliant II SYBR Green QPCR Master Mix, 80 ng cDNA, and 0.8 μL (10 μM) paired forward and reverse primers. The PCR program was performed for 40 cycles with denaturation 95°C for 30 s, followed by annealing and extension 60°C for 1 min. A dissociation step (95°C 15 s, 60°C 1 min, 95°C 15 s, and 60°C 15 s) was added to confirm the amplification specificity for each gene. Data were analyzed using the Applied Biosystems 7500 system software version 1.3.1, and relative copy numbers were computed according to the 2^−ΔΔCt^ method using a statistical confidence interval of 95%. To compare the differences between samples of the same gene, the gene expression values were standardized with the formula: (expression value of the gene – the average expression value of the same gene in different samples)/the standard deviation.

### Weighted Gene Coexpression Network Analysis (WGCNA) and Gene Ontology (GO) Analysis

The expression data generated by qRT-PCR were analyzed using the *WGCNA* R package (version 1.69) (Langfelder and Horvath, 2008) to detect modules comprising genes with similar expression patterns. A total of 3,950 genes in *P. falciparum* with higher variance (> 0.0001) were used for module detection. The weighted adjacency matrix was calculated based on Pearson’s correlation coefficients between gene pairs and soft-power threshold (β). The power (β) was set to 10 for high scale independence and low mean connectivity. The network type of “signed hybrid” was selected to construct the adjacency matrix. The topological overlap measure (TOM) matrix, which was transformed from the adjacency matrix, was used to estimate the connectivity in the network and was used for downstream analysis. By average linkage hierarchical clustering and a minimal size of 30 genes, all genes were classified into different gene modules based on similar expression profiles. Similar modules were merged using the maximum dissimilarity of modules set to 0.1. Other parameters were also set based on the data characteristics of this study. The different module eigengenes and developmental traits were then correlated (**Supplementary Figure 2**).

GO analysis of the genes in the module of interest was performed using *clusterProfiler* R package (version 3.16.1) (Yu et al., 2012). The cutoff *p*-value was set at 0.05.

### Recombinant Protein and Polyclonal Antibody Generation

The tagged genes of interest from PF3D7 chromosome 14 were cloned using the gateway technology with Clonase II (Invitrogen) according to the manufacturer’s instructions. Briefly, primers for each gene were designed using Primer BLAST to amplify the appropriate region of the proteins (the N-terminal putative signal peptides and transmembrane domains were avoided as much as possible to promote the hydrophilicity of the recombinant proteins) and then incorporated into the 25 bp-attB sites. The gene fragments were amplified from PF3D7 cDNA using high-fidelity Phusion DNA polymerase (Finnzymes Oy, Espoo Finland). PCR was performed using specific primers detailed in **Supplementary File 2**. The amplified product was purified using a DNA Gel Extraction Kit (Axygen, Union City, CA, USA) and then cloned into the entry plasmid pDONR221 by the BP recombination reaction. Positive clones were selected by transforming competent cells. Entry plasmids were then used to perform the LR recombination reaction to transfer the gene into the expression plasmid pDEST17. Positive expression clones in *E. coli* Transetta (DE3) (TransGen Biotech, Beijing, China) were selected by sequencing to obtain expression plasmids with the correct reading frame.

The recombinant proteins with His-tag were purified with Ni-NTA agarose (Qiagen) according to the manufacturer’s instructions. All proteins were analyzed by 12% sodium dodecyl-sulfate polyacrylamide gel electrophoresis (SDS-PAGE) and western blot with monoclonal antibodies against His-tag (**Supplementary Figure 3**, Cell Signaling Technology, Danvers, MA, USA). Rabbit polyclonal antibodies were prepared at Beijing Protein Innovation (Beijing, China) by immunizing New Zealand white rabbits with the recombinant proteins. Total IgG from sera of rabbits immunized with recombinant proteins was purified with the rProtein A Sepharose Fast Flow Kit (GE Healthcare, Chicago, IL, USA) according to the manufacturer’s instructions. Then the concentration of each IgG was adjusted to 10 mg/mL with PBS and aliquots of the antibodies were stored at -80°C.

### Western Blotting

Highly synchronized parasites of different developmental stages were obtained to detect the expression of selected proteins by western blotting. After lysis of infected RBCs with 10% saponin buffer, the parasites were collected by centrifugation at 1,800 × *g* for 10 min at 4°C and lysed with RIPA lysis and extraction buffer for 30 min on ice with Halt Protease Inhibitor Cocktail (both from Thermo Fisher Scientific). The protein mixture was centrifuged at 12,000 rpm for 30 min at 4°C and protein concentrations were quantified using the Pierce BCA kit (Thermo Fisher Scientific) in accordance with the manufacturer’s instructions. The extracted proteins were denatured by boiling with SDS-PAGE buffer, separated by 12% SDS-PAGE, and transferred to polyvinylidene difluoride membranes (Millipore, Bedford, MA, USA). The blots were blocked with a buffer containing 5% skimmed milk for 1 h at 25°C, and incubated in the same buffer with rabbit sera against merozoite proteins (1:1,000 dilution) or rabbit IgG control overnight at 4°C. After washing, detection was performed by incubation with the IRDye 800 CW conjugated goat anti-rabbit IgG (H+L) antibody (Li-COR Biosciences, Lincoln, NE, USA) using Odyssey Imaging System (Li-COR).

### Immunofluorescence Assay

Immunofluorescence assays were performed to locate the expression of the selected proteins in parasites at different stages. Thin smears of culture were fixed with ice-cold methanol for 10 min, penetrated with 0.01% Saponin (in PBS) and blocked with 10% bovine serum albumin (BSA) buffer for 30 min at room temperature. The samples were then incubated with a mouse IgG against merozoite surface protein (MSP-1, Thermo Fisher Scientific, 1 μg/mL) and rabbit IgG against merozoite proteins (1 μg/mL) or the IgG control derived from naïve rabbits for 60 min at 37°C, followed by staining with a goat-anti mouse IgG Alexa Fluor 488 and goat anti-rabbit IgG Alexa Fluor 555 (both from Invitrogen) for 60 min at 37°C. The nuclei were stained with 4, 6-diamidno-2-phenylindole (DAPI; Invitrogen). Fluorescence was visualized using a TCS SP5 confocal microscope (Leica Microsystems, Wetzlar, Germany).

### Detection of Antibodies in Sera From FM Patients

Ninety-six-well plates were coated (100 μL/well) with 1 μg/mL of the targeted proteins in coating buffer (Sigma-Aldrich) overnight at 4°C, with human IgG (1 μg/mL) and phosphate-buffered saline as positive and negative controls, respectively. Sera samples (100 μL from 1:100 dilution) were added to the wells pretreated with 10% skimmed milk. Goat anti-human polyvalent immunoglobulin (α-, γ-, and μ-chain specific) conjugated to alkaline phosphatase (Sigma-Aldrich) were used as secondary antibodies at a dilution of 1:10,000. The reaction was developed using p-nitrophenyl phosphate (Sigma-Aldrich) and stopped with 3 M sodium hydroxide. Optical density (OD) values were measured at 405 nm using a microplate reader. OD values from different plates were weighted by the OD value of human IgG at 0.1 μg/mL. Glutamate-rich protein (Glurp, XP_001347628.1, 916–930 aa, ILPEDKNEKVEHEIVEVEEILPEDKNEKGQ) coupled with BSA was used as a positive control. The cutoff value for positive results was set at 2.1-fold the mean OD value of sera samples from healthy individuals.

### Invasion Inhibition Assay

Synchronized schizont-stage parasites (200 μL) were added to each well of 96-well U-bottom plates with approximately 0.5% parasitemia and 1% hematocrit. The concentration of purified IgG was adjusted with PBS buffer. Afterwards, 5 μL of 4 mg/mL, 0.4 mg/mL and 0.04 mg/mL IgG was gently mixed to the indicated wells to obtain final concentration of 100 μg/mL, 10 μg/mL and 1 μg/mL, with replicates, 5 μL PBS was used as a control. Total IgG from one rabbit was used in each experiment and three independent experiments were carried out. The cultures were incubated in a candle jar at 37°C. After incubation for approximately 40 h, the cells were harvested and parasitemia was determined by flow cytometry as previously described (Hou et al., 2020). Briefly, 1–2 × 10^6^ cells were fixed with 1 mL 0.025% (v/v) glutaraldehyde at room temperature for 20 min and permeated with 0.5 mL 0.01% saponin at room temperature for 5 min. After staining with propidium iodide (PI, 10 μg/mL), the cells were detected and analyzed using a FACSCanto II flow cytometer (BD Biosciences, San Jose, CA, USA). The gate strategy and negative controls with uninfected RBCs were showed in **Supplementary Figure 5**.

### Statistical Analysis

The data were analyzed using Prism 5.0 (GraphPad, San Diego, CA, USA), and Excel 2010 (Microsoft, Redmond, WA, USA). The statistical significance of the experimental data was evaluated between two groups using two-tailed paired Student’s *t*-test or Mann-Whitney test, and among more groups using one-way analysis of variance. Statistical significance was set at *p* < 0.05.

## Results

### QRT-PCR-Based Transcriptome of Erythrocytic Parasites of *P. falciparum*

**Figure 1:**
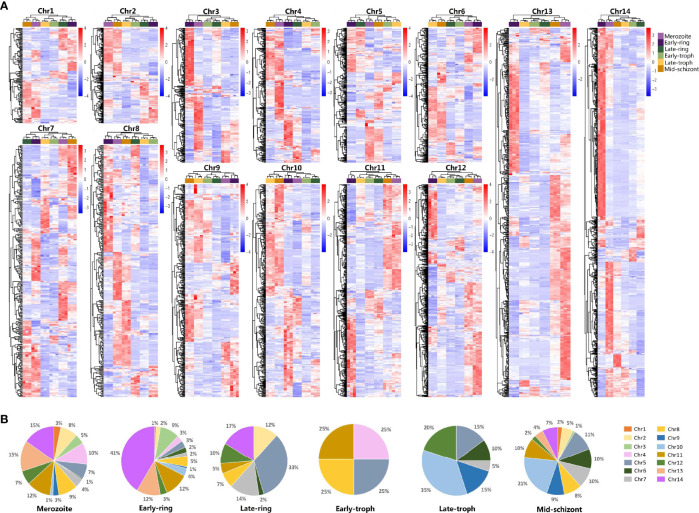
qRT-PCR analysis on the transcriptome profiles of *Plasmodium falciparum* during erythrocytic development. Different developmental stages of *P. falciparum* 3D7, including merozoite, early-ring, late-ring, early-trophozoite (early-troph), late-trophozoite (late-troph), and middle-schizont (mid-schizont), were collected. Transcription of genes in chromosomes 1–14 was detected by qRT-PCR. **(A)** Heatmaps of the transcriptome of each chromosome (chr). **(B)** Distribution of highly expressed genes (standard expression value >1, *p* < 0.05) at different developmental stages on chr1-14.

Using PlasmoDB data for PF3D7, 5,510 genes were obtained from the genomic sequences of chromosome 1–14. For further analysis, redundant sequences were removed (**Supplementary Table 2**). Specific primers for a total of 5,256 genes were successfully designed, of which 5,051 primers (**Supplementary Table 2**) effectively detected the expression of genes in the merozoite, early-ring, late-ring, early trophozoite, late-trophozoite, and middle-schizont stages by qRT-PCR. The collected data provide a developmental transcriptome of the erythrocytic process of *P. falciparum* (**Figure 1** and **Supplementary File 3**). The transcriptional features of the genes in each chromosome were showed in **Figure 1A**. Most of the highly expressed genes in the merozoite and early-ring stages mainly came from chr11-14, while chr5 had the most amounts of highly expressed genes in late-ring stage and chr10 in late-trophozoite and middle-schizont stages (**Figure 1B**). This indicated the genes on different chromosomes may be regulated by some coordinated regulatory mechanism.

### Characteristics of the Erythrocytic Developmental Transcriptome of *P. falciparum*

**Figure 2:**
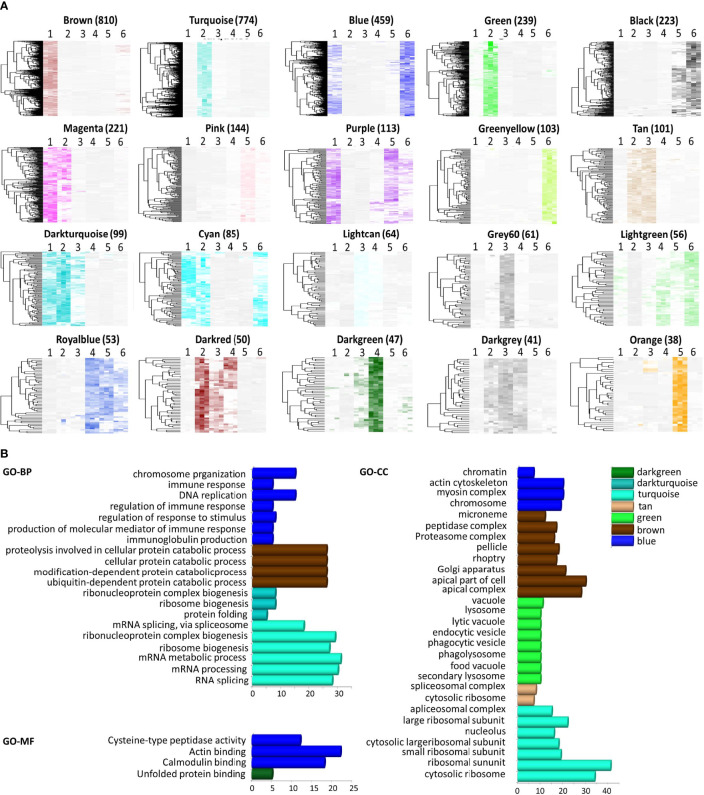
Module features and functional annotation of genes in the modules. Modules comprising genes with similar expression patterns were identified by weighted gene coexpression network analysis. **(A)** Heatmap of genes in each module (Number of genes was showed in parentheses). The samples were arranged as 1, merozoite, 2, early-ring, 3, late-ring, 4, early-trophozoite, 5, late-trophozoite, and 6, middle-schizont (three replicates for each stage). **(B)** Gene ontology (GO) enrichment analysis, including biological processes (BP), cellular component (CC), and molecular function (MF), for the genes comprised in each module.

To find the key modules associated with developmental stages, 3,950 genes in *P. falciparum* with higher variance (> 0.0001) were classified into different modules according to WGCNA, with β = 10 to guarantee high scale independence (near 0.9) and low mean connectivity (near 0). The dissimilarity of the modules was set as 0.1, and a total of 20 modules were generated (**Figure 2A** and **Supplementary File 4**). The clustering dendrograms of the samples matched the strip chart (**Supplementary Figure 1C**). The topology matrix was clustered using the dissimilarity between the genes, and then divided into different modules. Overall, 20 modules were identified. The numbers and heatmaps of the genes in each module are shown in **Figure 2A**. Next, a module and sample trait correlation heatmap was created based on correlations between module eigengenes and developmental stage traits (**Supplementary File 4** and **Supplementary Figure 2**). The orange module associated with late-trophozoite stage (average correlation, cor = 0.54), the green-yellow module mainly associated with late-schizont stage (cor =0.53), turquoise module associated with early ring stage (cor = 0.52), the brown module mainly associated with the merozoite stage (cor = 0.49), the grey60 module associated with late ring stage (cor = 0.49), and the green module associated with early ring stage (cor = 0.49) were the top six deepest modules (*p* < 0.05). Brown module (associated with merozoite) comprise 810 genes, the largest number of genes among the identified modules, followed by the turquoise module (associated with early ring) with 774 genes, and the blue module (associated with both middle schizont and merozoite) with 459 genes (**Figure 2A** and **Supplementary File 4**). These results indicated that a large number of genes were upregulated from middle schizont to early ring, whereas from late ring to late trophozoite stages no such violent fluctuation in gene expression was observed.

To reveal the significance of the gene expression variability observed, functional annotation of the module genes was performed by GO analysis. The genes were classified into three groups according to their involvement in biological processes, molecular functions, and cellular components (**Figure 2B**, **Supplementary File 5**). Genes in the turquoise module were mainly associated with RNA splicing, ribonucleoprotein complex biogenesis, ribosome biogenesis, mRNA processing, ribosomal subunit, cytosolic ribosome, among others processes. Genes in the blue module were associated with chromosome organization, DNA replication, immune system process, actin cytoskeleton, myosin complex, actin binding, and calmodulin binding. Genes in the brown module were associated with cellular protein catabolic process, modification-dependent macromolecule catabolic process, apical part of cell, apical complex, Golgi apparatus, among other mechanisms. Within these modules, genes in the brown modules were mostly associated with merozoites, and GO analysis indicated that these genes may be apical complex components, which are closely related to parasite invasion. Thus, the genes in the brown module were chosen for further investigations.

### Generation of Merozoite Proteins Encoded by Genes From Chromosome 14 of *P. falciparum*

A total of 810 genes were involved in the brown module, including 142 genes from chromosome 14 (**Supplementary File 5**). Among the chromosomes of *P. falciparum*, chromosome 14 is the only that does not contain variant antigen genes and is also the one with the highest number of genes (Gardner et al., 2002). Among the **142** coding genes of chromosome 14, 108 genes were unknown in function. To study the properties of proteins encoded by the potentially merozoite-associated genes of chromosome 14, recombinant proteins were generated. Finally, 12 proteins, encoded by four previously characterized genes and eight unknown genes, were successfully expressed. A flowchart indicates the strategy analysis of these 12 merozoite proteins was showed in **Supplementary Figure 1**. Information on these genes is listed in **Table 1**. The eight unknown proteins were named as *P. falciparum* merozoite protein (*Pf*MP) 209 (PlasmoDB gene ID: PF3D7_1414400), *Pf*MP344 (PF3D7_1434400), *Pf*MP373 (PF3D7_1437300), *Pf*MP409 (PF3D7_1440900), *Pf*MP442 (PF3D7_1444200), *Pf*MP587 (PF3D7_1458700), *Pf*MP614 (PF3D7_1461400), and *Pf*MP723 (PF3D7_1472300). All 12 recombinant proteins were analyzed by SDS-PAGE and confirmed by western blotting with an anti-His tag mouse monoclonal antibody (**Supplementary Figure 3**).

**Table 1:** Successfully expressed proteins encoded by the brown module genes in chromosome 14 of *Plasmodium falciparum* 3D7 strain.

Protein Name	PlasmoDB Gene ID	NCBI Reference Sequence ID	Predicted Molecular Weight
Serine/threonine protein phosphatase (PP1)	PF3D7_1414400	XM_001348279.1	34.9 kDa
NADP-specific glutamate dehydrogenase (GDH1)	PF3D7_1416500	XM_001348301.1	52.5 kDa
*Pf*MP209	PF3D7_1420900	XM_001348340.1	46.7 kDa
ERAD-associated E3 ubiquitin-protein ligase (HRD1)	PF3D7_1422500	XM_001348353.1	59.8 kDa
*Pf*MP344	PF3D7_1434400	XM_001348463.1	33.7 kDa
*Pf*MP373	PF3D7_1437300	XM_001348491.1	25.5 kDa
*Pf*MP409	PF3D7_1440900	XM_001348526.1	28.3 kDa
*Pf*MP442	PF3D7_1444200	XM_001348558.1	25.0 kDa
*Pf*MP587	PF3D7_1458700	XM_001348698.1	42.1 kDa
*Pf*MP614	PF3D7_1461400	XM_001348724.1	37.0 kDa
*Pf*MP723	PF3D7_1472300	XM_001348829.1	64.4 kDa
Acyl-CoA binding protein (ACBP)	PF3D7_1477800	XM_001348887.1	10.7 kDa

ERAD, endoplasmic reticulum-associated degradation; NADP, nicotinamide adenine dinucleotide phosphate; NCBI, National Center for Biotechnology Information; PfMP, Plasmodium falciparum merozoite protein.

### Expression Analysis of the Newly Identified Merozoite-Associated Proteins of *P. falciparum*

**Figure 3:**
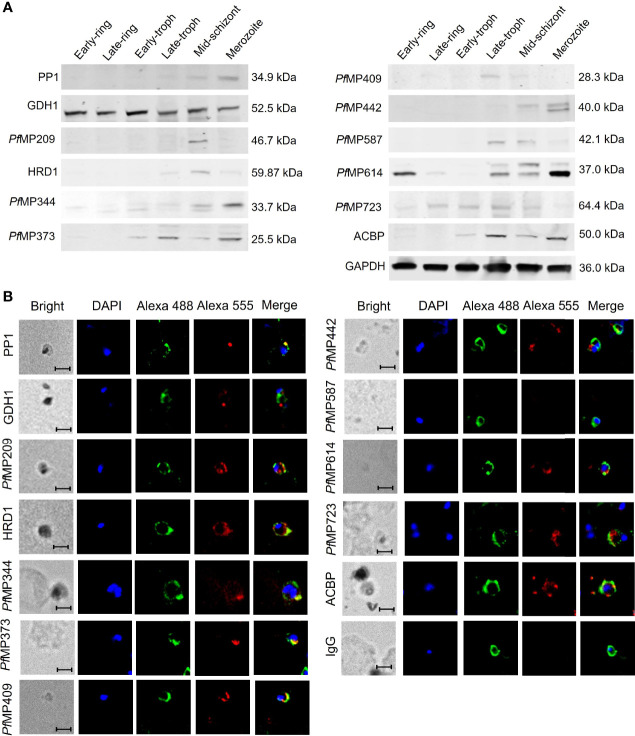
Expression of merozoite-associated proteins during the erythrocytic developmental stages of *Plasmodium falciparum*. **(A)** Expression of merozoite proteins in the six erythrocytic stages of *P. falciparum* 3D7 detected by western blot with total IgG from rabbit sera against each protein and glyceraldehyde-3-phosphate dehydrogenase B (GAPDH) as an internal reference. **(B)** Localization of the merozoite proteins in the merozoites shown by immunofluorescence. All samples were incubated with total IgG from rabbit sera against each merozoite protein or IgG control derived from naïve rabbits, total IgG from mice sera against MSP-1 were used for co-location. Alex Flour 555 donkey anti-rabbit IgG (red fluorescence) and Alex Flour 488 donkey anti-mouse IgG (green fluorescence). Green, anti-MSP1; Red, anti-individual candidate protein as indicated. Nuclei are stained with DAPI (blue). Scale bar: 5 μm. PP1, serine/threonine protein phosphatase 1; GDH1, nicotinamide adenine dinucleotide phosphate-specific glutamate dehydrogenase; *Pf*MP, *P. falciparum* merozoite protein; HRD1, endoplasmic reticulum-associated degradation-associated E3 ubiquitin-protein ligase 1; ACBP, acyl-CoA binding protein; MSP-1, merozoite surface protein-1.

The results of qRT-PCR showed the transcriptional expression of the genes encoding the merozoite-associated proteins was highest in merozoites, compared to that of other developmental stages (**Supplementary Figure 4**). Analysis of the expression of the 12 merozoite proteins using polyclonal antibodies showed that the expression patterns of these proteins varied significantly (**Figure 3A**). The molecular weights of most proteins are similar to those as predicted, except for *Pf*MP442 and ACBP, which are higher than predicted. PP1, *Pf*MP344, and *Pf*MP442 were highly expressed in the merozoites. In particular, the level of *Pf*MP614 increased from late-trophozoite, peaked in merozoites, and remained till the early ring stage. Moreover, *Pf*MP373 and ACBP showed highly expression from the early trophozoite to merozoite stage, and GDH1 levels were relatively stable throughout the erythrocytic stages. The other five proteins were expressed at low levels in merozoites. The localization of the 12 proteins was detected by immunofluorescence in merozoites (**Figure 3B**). Some of the merozoite proteins, including PP1, HRD1, *Pf*MP344, *Pf*MP373 and *Pf*MP409 were partially co-located with MSP-1, indicating these proteins may locate on the surface of merozoites. The other proteins were mainly expressed in the cytoplasm of merozoites, except for *Pf*MP587, which was not detected in merozoites.

### Low Antigenicity of Merozoite Proteins

**Figure 4:**
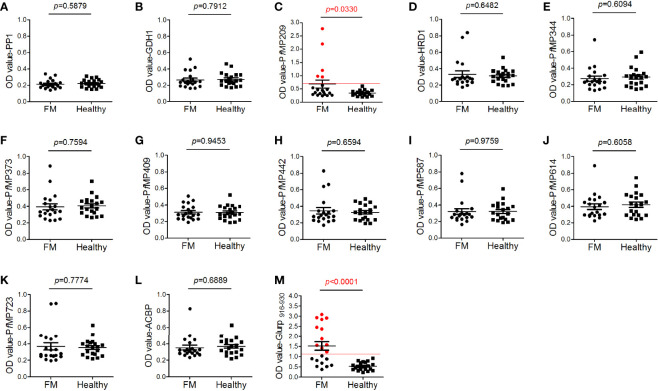
Antigenicity of merozoite proteins. Human serum antibody levels against the merozoite proteins, including serine/threonine protein phosphatase 1 [PP1, **(A)**], nicotinamide adenine dinucleotide phosphate (NADP)-specific glutamate dehydrogenase [GDH1, **(B)**], *Plasmodium falciparum* merozoite protein (*Pf*MP)209 **(C)**, endoplasmic reticulum-associated degradation (ERAD)-associated E3 ubiquitin-protein ligase 1 [HRD1, **(D)**], *Pf*MP344 **(E)**, *Pf*MP373 **(F)**, *Pf*MP409 **(G)**, *Pf*MP442 **(H)**, *Pf*MP587 **(I)**, *Pf*MP614 **(J)**, *Pf*MP723 **(K)**, acyl-CoA binding protein [ACBP, **(L)**], and glutamate-rich protein (Glurp, 915–930aa, **M**), were determined by ELISA. A total of 20 sera from patients with falciparum malaria (FM) and 20 sera from healthy individuals (healthy) were tested. Red lines indicate the cutoff values of the positive tests (≥ 2.1-fold the mean optical density value of the healthy group). Red dots indicate positive individuals. Data are presented as the median ± standard deviation.

To study the antigenicity of the newly identified merozoite-associated proteins, ELISA was performed to test the antibodies against merozoite proteins in the sera of FM patients. The epitope Glurp_915–930_, which has high antigenicity (Hou et al., 2020), coupled with BSA, was used as a positive control. Among all the 12 merozoite proteins, only the antibodies against *Pf*MP209 in the sera of FM patients were at levels significantly higher than those in healthy individuals (**Figure 4C**). The peptide of Glurp (915-930aa) had higher level of antibodies in the sera of FM and more than half of the patients were serologically positive (**Figure 4M**). In contrast, only 25% of patients were serologically positive for *Pf*MP209. The OD value of antibodies to Glurp_915–930_ in FM sera was also much higher than that of antibodies to *Pf*MP209 (Glurp_916–930_ vs. *Pf*MP209, 1.53 ± 0.93 vs. 0.683 ± 0.679, *p* = 0.0022). Overall, the levels of the antibodies against all merozoite proteins, except for Glurp_915–930_, were very low (**Figure 4**), indicating the low antigenicity of these merozoite proteins.

### Antibodies Specific for the Newly Identified Merozoite-Associated Proteins Interfered With Parasite Invasion

**Figure 5:**
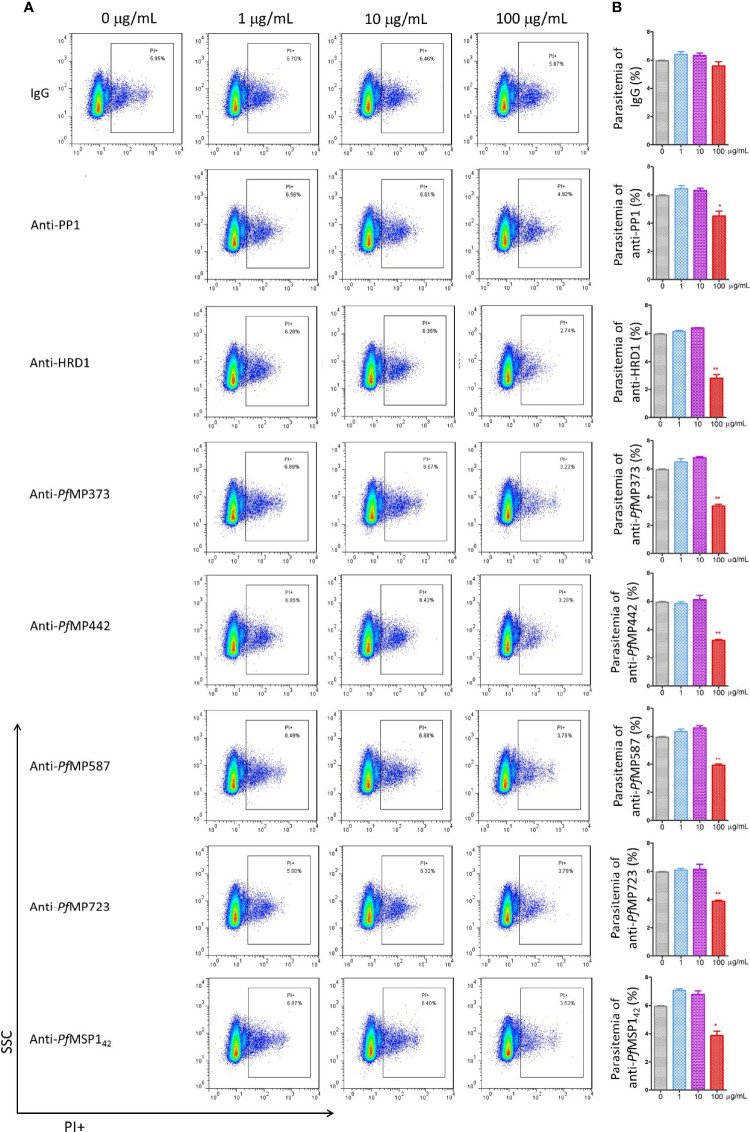
Merozoite protein-specific antibodies inhibit parasite proliferation. Highly synchronized schizont-stage parasites of *P. falciparum* 3D7 strain were cultured in the presence of total IgG containing polyclonal antibodies (Ab) against merozoite proteins and IgG control derived from naïve rabbits. Parasitemia was determined using flow cytometry after 40–42 h of culture. **(A)** Representative dot plots showing the frequency of propidium iodide positive infected red blood cells (iRBCs). **(B)** Histograms comparing parasitemia between the antibody-treated group and the IgG control group using Mann-Whitney test. These results are representative of three independent experiments, with data indicating mean ± standard deviation. *p < 0.05 and **p < 0.001. *Indicates comparison with the corresponding naïve IgG group. PP1, serine/threonine protein phosphatase 1; HRD1, endoplasmic reticulum-associated degradation (ERAD)-associated E3 ubiquitin-protein ligase 1; *Pf*MP, *Plasmodium falciparum* merozoite protein; MSP-1_42_, merozoite surface protein-1 42 kDa fragment.

To determine whether the merozoite protein-specific antibodies could be protective, invasion inhibition assays were performed to investigate the neutralization effect of the total IgG from the immunized rabbits, using antibodies against MSP-1_42_ as positive control. Antibodies specific for PP1, HRD1, *Pf*MP373, *Pf*MP442, *Pf*MP587, *Pf*MP723, and MSP-1_42_ showed an invasion inhibitory effect of over 30% compared with the control IgG group at a concentration of 100 μg/mL (**Figure 5**). Antibodies specific for GDH1, *Pf*MP409, *Pf*MP614, and ACBP showed relatively low inhibitory effects, and those specific for *Pf*MP209 and *Pf*MP344 had no effect (**Supplementary Figure 6**).

## Discussion

*Plasmodium falciparum* parasites have compact genomes that regulate their morphological and biological diversity. However, the function of more than 40% of the genes remains unknown (Gardner et al., 2002), which hampers the development of effective drugs and vaccines. Our study analyzed the gene expression landscape during parasite growth and development within erythrocytes, aiming to discover functionally important genes.

Tremendous work have been done on transcriptome screening of *P. falciparum* with various postgenomic methods, such as RNA-sequence (Toenhake et al., 2018; Wichers et al., 2019) or microarray (Bozdech et al., 2003; Daily et al., 2005). Compared to these two methods, qRT-PCR is more accurate, for targeted sequencing may be influenced by amplification preference during the construction of sequencing libraries and the accuracy of gene chip analysis may be influenced by the complicated operation process including probe labeling, chip printing and hybridization. Thus, qRT-PCR is routinely used to validate the results of sequencing and gene chip anlaysis. Therefore, the transcription of more than 5,000 genes distributed on the 14 chromosomes of PF3D7 strain was herein examined by qRT-PCR. A qRT-PCR-based transcriptome was obtained, thereby providing a global view of the gene expression profile throughout the erythrocytic cycle of *P. falciparum*, which may pave the way for a better understanding of the parasite biology and represent a reference dataset.

WGCNA provided clusters enriched for genes involved in specific biological processes and displayed distinct patterns of expression throughout the erythrocytic cycle of *P. falciparum*. The majority of the gene clusters showed predominant expression in specific stages, offering new guidance as to where and how these genes may function. Overall, the parasites upregulate a large number of genes from the stage of merozoite release to invasion into new erythrocytes, until the parasites settle down in the erythrocytes, whereas gene expression remains relatively stable during the intra-erythrocytic developmental stage. This increase in gene expression indicates that the parasite mobilizes several gene resources to invade erythrocytes. Furthermore, some overlap of upregulated genes was observed among the middle-schizont, merozoite, and early ring stages, but a considerable number of genes were upregulated in only one of these three periods, indicating that the entire process of invasion is complicated and genes responsible for merozoite release, invasion, and settlement are different.

Totally, 135 genes of chromosome 14 within the brown module, which was associated with merozoites, were selected for further investigation. Overall, 12 potentially merozoite-associated proteins encoded by these genes were successfully generated. Within the four known proteins herein identified, *Pf*PP1 has a highly conserved PP1 catalytic subunit, similar to other PP1 members (Kumar et al., 2002; Pierrot et al., 2018; Zeeshan et al., 2021). It was reported that *Pf*PP1 is expressed in all erythrocytic stages of *P. falciparum* parasites (Kumar et al., 2002), and our results showed that *Pf*PP1 is highly expressed in merozoites and its expression is low in other erythrocytic stages. Noteworthy, abrogation of PP1 expression by synthetic short interfering RNA was shown to lead to the inhibition of parasite DNA synthesis (Pierrot et al., 2018). Our results showed that antibodies specific to *Pf*PP1 can interfere with the growth and proliferation of the parasites *in vitro*. The malarial enzyme *Pf*GDH1 was found to be present in the plasma of patients with acute *P. falciparum* infection (Rodriguez-Acosta et al., 1998). Moreover, *Pf*GDH1 has been studied and regarded as a rational target of new antimalarial drugs, as it plays key roles in cellular redox, amino acid, and energy metabolism (Krauth-Siegel et al., 1996; Wagner et al., 1998; Zocher et al., 2012). Herein, *Pf*GDH1 was found stably expressed throughout the erythrocytic process, and the antibodies specific to recombinant *Pf*GDH1 showed a slight inhibitory role in the *in vitro* assays. Endoplasmic reticulum-associated degradation (ERAD)-associated E3 ubiquitin-protein ligase is the central component of the ERAD machinery and causes the ejection of the misfolded substrate into the cytosol for efficient proteasomal degradation by coordinating with multiple endoplasmic reticulum luminal and cytosolic adaptors (Smith et al., 2011). However, to date, there has been no report on the ERAD-associated E3 ubiquitin-protein ligase HRD1 of *Plasmodium*. Our study showed that *Pf*HRD1 is more expressed in middle schizonts than in the other five erythrocytic stages, and that *Pf*HRD1 specific antibodies can significantly reduce parasitemia by more than half *in vitro* (100 μg/mL, 5.95% ± 0.08% vs. 2.82% ± 0.45%; *p* = 0.0003). *Pf*ACBPs are globular-helical proteins containing a conserved acyl-CoA binding region and the critical targets of the antimalaria drug mefloquine, which competitively inhibits fatty acyl-CoA binding to *Pf*ACBPs, thereby leading to the prevention of *P. falciparum* growth and proliferation (Karmodiya and Surolia, 2006). *Pf*ACBP was found to be highly expressed in the second half of the erythrocytic growth of the parasite, with antibodies specific to *Pf*ACBP being able to induce a slight reduction in parasite proliferation.

Within the unknown proteins herein identified, *Pf*MP209 was annotated as mannose-1-phosphate guanyltransferase, *Pf*MP587 as exonuclease V, *Pf*MP344 and *Pf*MP723 as membrane proteins, and the others as conserved *Plasmodium* proteins. Treatment with antibodies specific to *Pf*MP373, *Pf*MP442, *Pf*MP587, and *Pf*MP723 showed a reduction of more than 30% parasitemia *in vitro*, but antibodies specific to *Pf*MP209, *Pf*MP344, *Pf*MP409, and *Pf*MP614 were less effective or ineffective in inhibiting parasite growth. Hence, these novel merozoite-associated proteins represent potential targets for the development of new antimalarial drugs or vaccines.

Our previous work revealed that immunogenic epitopes or antigens of malaria parasites may serve as decoy epitopes to attract host humoral immunity to protect the functional domains of the antigens (Hou et al., 2020). Interestingly, in this study, although antibodies against more than half of the 12 merozoite-associated proteins identified could interfere with the parasite proliferation, the levels of the antibodies against these proteins was very low in FM patients, indicating that these proteins may have low antigenicity. These results further support the idea that malaria functional proteins or domains, which play critical roles in merozoite proliferation and invasion, are less to be recognized and attacked by the host humoral immunity.

In conclusion, the qRT-PCR-based transcriptome of erythrocytic parasites of *P. falciparum* herein described provides new insights into gene function in the parasite developmental progression. Based on the transcriptomic data, a cluster of genes associated with merozoites was discovered, and several novel merozoite-associated proteins were found to play important roles in the invasion process of *P. falciparum*. However, the specific pathogenic mechanisms of these newly identified merozoite-associated proteins remain unclear and require further investigations.

## Data Availability Statement

The original contributions presented in the study are included in the article/**Supplementary Material**. Further inquiries can be directed to the corresponding authors.

## Ethics Statement

The studies involving human participants were reviewed and approved by the Ethical Committee of the Chinese Academy of Medical Sciences. The patients/participants provided their written informed consent to participate in this study.

## Author Contributions

NH and QC conceived and designed experiments. NH, SLi, and NJ performed the majority of the experiments. YM, XP, and SLiu performed some experiments. NH and QC analyzed the data and wrote the manuscript. All authors reviewed the results and approved the final version of the manuscript.

## Funding

This work was supported by CAMS Innovation Fund for Medical Sciences (CIFMS) [Grant Number 2021-1-I2M-038 and 2019-I2M-5-042], the National Natural Science Foundation of China [Grant Numbers. 81672050, 81420108023, 82030060], National Science and Technology Major Project [Grant Numbers 2018ZX10101001].

## Conflict of Interest

The authors declare that the research was conducted in the absence of any commercial or financial relationships that could be construed as a potential conflict of interest.

## Publisher’s Note

All claims expressed in this article are solely those of the authors and do not necessarily represent those of their affiliated organizations, or those of the publisher, the editors and the reviewers. Any product that may be evaluated in this article, or claim that may be made by its manufacturer, is not guaranteed or endorsed by the publisher.

## Supplementary Material

Supplementary File 1 Primers for qRT-PCR.

Supplementary File 2 Primers and amino acid sequences for clone construction.

Supplementary File 3 qRT-PCR data.

Supplementary File 4 Module information and correlation analysis.

Supplementary File 5 Gene Ontology analysis of genes within the modules.

Supplementary File 6 WB images of **Figure 3A**.
