# Supplementary material for: Merozoite Proteins Discovered by qRT-PCR-Based Transcriptome Screening of Plasmodium falciparum
Source: Front Cell Infect Microbiol. 2021 Dec 9;11:777955. doi: 10.3389/fcimb.2021.777955 (PMC8696357; doi:10.3389/fcimb.2021.777955)
Supplement: Supplementary File 1 — Primers for qRT-PCR. [file DataSheet_1.pdf]

Supplementary Materials and Methods

Animals and sample collection

We used wild type, Apc$^{+/1638N}$ (1), *vil*-TRα1 and *vil*-TRα1/Apc$^{+/1638N}$ animals (2). Animals were housed in the same animal facility and received standard mouse chow and water *ad libitum*. All experiments were performed in compliance with the French and European guidelines for experimental animal studies and approved by local and national ethics committees (agreement 02847.01). Animals were sacrificed, and the intestine (normal mucosae and tumors) was quickly removed. We recovered tumors and normal portions of the mucosa under a binocular microscope (Olympus) and froze them in liquid nitrogen for RNA extraction or the ChIP assay. For this study, normal intestine and tumors were from the proximal small intestine.

Cell lines and transfection experiments

This study was performed on the human Caco2 colorectal cancer cell line (from the American Type Culture Collection). Caco2 cells (50,000 cells/well in 24 multiwell plates) were cultured in DMEM supplemented with 10% heat- inactivated fetal calf serum. We used the following vectors: pGl2-DR4-Luc and pGl2- mutDR4-Luc (negative control for TR activity. 200 ng/well) and pGS5-TRα1 (3) (100 ng/well); TopFlash and FopFlash (negative control for WNT activity. Upstate, 200 ng/well); pCIneo-β-cateninXL (100 ng/well), Evr2-Tcf4E (100 ng/well; kind gift of Professor Waterman, UC Irvine, USA); pRL-CMV (1 ng/well; Promega). Data from experiments performed with mutDR4-Luc and FopFlash are not shown. The vectors were transfected using the Exgen transfection reagent (Euromedex). Luciferase activity was measured 48 hours after transfection using the luciferase dual system (Promega).

RNA extraction and RTqPCR analysis

RNA was extracted from tissue samples using the Qiagen RNeasy Kit (Qiagen). To avoid the presence of contaminating DNA, DNase digestion was performed on all preparations. Reverse transcription was performed using MuMLV reverse transcriptase (Promega) on 1 µg of total RNA using random hexanucleotide priming (Promega) according to the manufacturer's instructions. For the primary cultures, RNA was extracted using the Absolutely RNA Nanoprep Kit (Stratagene). Reverse transcription was performed using the SuperScript III First-Strand Synthesis SuperMix for qRT-PCR (Invitrogen) on 300 ng of total RNA. All of the cDNA samples were purified using the Qiagen PCR Purification Kit (Qiagen) before use for the qPCR experiments. The qPCR was performed with SYBR green PCR master mix (Qiagen) in an MxP3000 apparatus (Stratagene). The data from the qPCR were normalized to Ppib levels for each sample. The primers sequences are available upon request.

Chromatin immunoprecipitation (ChIP)

The ChIP study was performed on 10 mg of samples collected from the intestine of vil-TRα1/Apc (healthy mucosa and tumors) and WT mice. The samples were minced with a razor blade and crosslinked with 1% formaldehyde at room temperature for 15 minutes. The crosslinking reaction was stopped by the addition of glycine to a final concentration of 0.125 M, and the samples were incubated at room temperature for 5 minutes. The samples were then centrifuged at 200 g to pellet the pieces. The fragments were washed once in cold PBS containing protease inhibitor cocktail (Roche) and then disaggregated by 10 strokes in a Dounce homogenizer. The resulting homogenate was centrifuged at 2000 g, the pellet recovered was then incubated in SDS buffer (1% SDS, 50 mM TRIS pH 8.1, 10 mM EDTA) on ice for 10 minutes. After 15 minutes of sonication (30 sec on/30 sec off cycles, Biodisruptor), the samples were centrifuged at maximum speed at 4°C, and the supernatant was recovered and quantified by Nanodrop (ND-1000 UV-Vis Spectrophotometer, NanoDrop Technologies). The same amount of each sample was diluted 1:10 in dilution buffer (0.01% SDS,

1.1% Triton X-100, 1.2 mM EDTA, 16.7 mM Tris-HCl, pH 8.1, 167 mM NaCl). After preclearing with Protein A magnetic beads (Invitrogen), each sample was divided into four samples, and the precipitation was performed using 9 µg of each antibody (anti-Tcf4, Santa Cruz; anti β-catenin, Santa Cruz; anti-TRα1 (3) or rabbit IgG). At the end of the reaction and washing steps, the complexes were recovered using protein A magnetic beads. The DNA was extracted after Proteinase K (Fermentas) and phenol-chloroform treatments. Specific DNA fragments were analyzed by qPCR using a SYBR green PCR master mix (Qiagen) in an MxP3000 apparatus (Stratagene). The primers designed to amplify the genomic regions containing: 1) TREs we previously described (3,4); 2) WREs described in literature (5,6); and 3) the *Villin* and *Rplp0* (36B4) promoters, as negative controls (3). The *Ppia* gene was used in all reactions as internal control. Primer sequences are available upon request.

Statistical Analysis

Statistical analysis was performed by using unpaired Student's t-tests and the software GraphPad Prism (version 8; GraphPad Software Inc., San Diego, Calif., USA). The level of significance was established at $p < 0.05$.

References

1. Fodde R, Edelmann W, Yang K, Van Leeuwen C, Carlson C, Renault B, Breukel C, Alt E, Lipkin M, Khan PM, et al. A targeted chain-termination mutation in the mouse Apc gene results in multiple intestinal tumors. *Proc Natl Acad Sci U S A* (1994) **91**:8969–8973. doi:10.1073/pnas.91.19.8969
2. Kress E, Skah S, Sirakov M, Nadjar J, Gadot N, Scoazec JY, Samarut J, Plateroti M. Cooperation Between the Thyroid Hormone Receptor TRα1 and the WNT Pathway in the Induction of Intestinal Tumorigenesis. *Gastroenterology* (2010) **138**:1863-1874.e1. doi:10.1053/j.gastro.2010.01.041
3. Plateroti M, Kress E, Mori JI, Samarut J. Thyroid Hormone Receptor α1 Directly Controls Transcription of the β-Catenin Gene in Intestinal Epithelial Cells. *Mol Cell Biol* (2006) **26**:3204–3214. doi:10.1128/mcb.26.8.3204-3214.2006
4. Kress E, Rezza A, Nadjar J, Samarut J, Plateroti M. The frizzled-related sFRP2 gene is a target of thyroid hormone receptor α1 and activates β-catenin signaling in mouse intestine. *J Biol Chem* (2009) **284**:1234–1241. doi:10.1074/jbc.M806548200
5. Mahmoudi T, Boj SF, Hatzis P, Li VSW, Taouatas N, Vries RGJ, Teunissen H, Begthel H, Korving J, Mohammed S, et al. The leukemia-associated Mllt10/Af10-Dot1l are Tcf4/β-catenin coactivators essential for intestinal homeostasis. *PLoS Biol* (2010) **8**: doi:10.1371/journal.pbio.1000539
6. Hu MC, Rosenblum ND. Smad1, β-catenin and Tcf4 associate in a molecular complex with the Myc promoter in dysplastic renal tissue and cooperate to control Myc transcription. *Development* (2005) **132**:215–225. doi:10.1242/dev.01573
